# Revisiting the relationship between maternal parenting behaviors and executive functions in young children: Effect of measurement methods

**DOI:** 10.3389/fpsyg.2023.985889

**Published:** 2023-03-14

**Authors:** Wei Wei, Wen-Ting Lu, Min-Min Huang, Yan Li

**Affiliations:** ^1^Shanghai Institute of Early Childhood Education, Shanghai Normal University, Shanghai, China; ^2^Early Child Development Research Center, Shanghai Normal University, Shanghai, China

**Keywords:** maternal parenting, executive functions, measurement method, reported parenting, observational parenting, preschoolers

## Abstract

The past decade of studies showed that parenting behaviors (e.g., warmth, autonomy, and control) were associated with children’s executive functions (EF) in the early years. However, different measurement methods had been used across studies, making it hard to compare the effects of parenting on EF across studies. Therefore, the present study aimed to examine the effect of the measurement methods on the relationship between maternal parenting behaviors and children’s EF among a group of Chinese preschoolers. One hundred and twenty-six children (62 boys; Mage = 48.65 months) were assessed with direct measures on children’s EF (inhibition and working memory tasks), and parenting behaviors of their mothers during interaction with children were observed and coded. Mothers also reported their parenting practices and children’s difficulties in executive functions. The results of structural equation modeling showed that the latent performance-based EF was uniquely predicted by maternal positive control and negative control in mother–child interaction, while children’s EF difficulties reported by mothers were predicted by mother-reported warmth and support, and autonomy granting. Overall, the results suggest that the relationship between maternal parenting and children’s EF depends on the measurement methods of parenting and executive functions.

## 1. Introduction

Executive functions (EF) is typically described as a group of cognitive skills (e.g., working memory, inhibition, attentional shifting, and planning) that engage in goal-directed activities and regulate the cognitive, emotional, and behavioral processes (e.g., Anderson, 2002; Best et al., 2009). It is well established that EF is related to a wide range of developmental outcomes such as academic achievement and social competence (for a review, see Zelazo et al., 2016). EF grows rapidly in the early years (Garon et al., 2008) and is shaped by the early family environment, especially the parenting behaviors.

Parenting typically included different aspects derived from responsiveness and control (Baumrind, 1967; Maccoby and Martin, 1983). Responsiveness refers to parents’ appropriate, timely, contingent responses to children’s needs and feelings (Landry et al., 2006), while control refers to the parents’ efforts to regulate children’s behavior, either in a positive way such as scaffolding or in a negative way such as punishment (Roskam et al., 2014; Rhee et al., 2015). Parental responsiveness is argued to provide emotional support for children and help the development of parent–child attachment, with which children can explore the environment actively and practice their skills (Birmingham et al., 2017). Positive control, such as scaffolding and cognitive instructions, provides external regulation of children’s emotional and behavioral processes and helps children internalize the regulation strategies (Bernier et al., 2012). In contrast, negative control such as corporal punishment and over-controlling may undermine the parent–child relationship as well as children’s autonomy, and thus have negative influence on children’s cognitive skills.

The relations between three aspects of parenting behaviors and children’s EF have been well established in previous studies (e.g., Bernier et al., 2010, 2012; Blair et al., 2014; Eisenberg et al., 2015; Lucassen et al., 2015; Merz et al., 2016; Hertz et al., 2019; Regueiro et al., 2020; for a review, see Fay-Sambac et al., 2014). In a meta-analysis study, Valcan et al. (2018) reported children’s global EF significantly related to positive (e.g., warmth, responsiveness; *r* = 0.20), negative (e.g., intrusiveness, detachment; *r* = −0.22), and cognitive parenting behaviors (e.g., autonomy support, scaffolding; *r* = 0.20). However, the relationship may vary across studies with different measurements of parenting or EF.

Parenting behaviors can be measured by questionnaire reports in which parents rate their daily behaviors in various family situations, or by a direct observation method in which the experimenters rate the parenting behaviors during parent–child interaction. Both measurement methods have their methodological advantages and disadvantages. For example, questionnaires can include assessment on parents’ behaviors in a variety of situations and over time, but parents’ responses may suffer from a social-desirability bias (Zahidi et al., 2019). In contrast, observation is more objective, but the parents’ behaviors may be specific to some contexts and suffer from observer effects (Gardner, 2000). Although both self-reported and observational parenting can assess the same parenting constructs, some studies found little relationship between the self-reported and observational parenting even for the identical parenting aspect (e.g., Bennett et al., 2006; Herbers et al., 2017; Zahidi et al., 2019). For example, Herbers et al. (2017) reported that neither positive parenting nor negative parenting of young children’s parents assessed with self-reported and observation methods correlated with each other. Furthermore, a recent meta-analysis study by Hendriks et al. (2018) indicated only an average correlation of 0.17 between parent-reported and observational parenting.

Similarly, children’s EF can be assessed with different measures. The first one is a direct evaluation of children’s performance in cognitive tasks tapping EF subcomponents, such as laboratory tests for inhibition (such as Stroop tasks, asking children to report the required information and ignore others) and working memory (such as backward number span, asking children to recall information in reversed order). Even though numerous measures for each EF subcomponent were developed for young children, some studies (e.g., Willoughby et al., 2010; Wiebe et al., 2011) using factor analysis showed that measures for different EF subcomponents were loaded on a single common EF factor during the early years. Another method is questionnaire measures by asking parents or teachers to report children’s everyday problem behaviors related to EF. For example, the widely used Childhood Executive Functioning Inventory (Thorell and Nyberg, 2008) assesses children’s inhibition, regulation, working memory, and planning. Unlike the underlying EF skills measured by the laboratory tests, questionnaires may assess application of EF skills in real world (Thorell and Nyberg, 2008). Although both laboratory tests and questionnaires putatively measure children’s EF, recent studies showed that children’s scores on both measures may not correlate with each other (e.g., Buchanan, 2016; Saunders et al., 2018; Eisenberg et al., 2019). For example, Saunders et al. (2018) reanalyzed five datasets involving about 2,600 participants, and the results showed that reported inhibition, one aspect of EF, did not correlate with their performance on laboratory tasks of inhibition (Stroop and Flanker). In one meta-analysis study, Toplak et al. (2013) included about 300 correlations between performance-based and reported EF in 20 studies, but found that only 24% were statistically significant, and the median of the correlations was only 0.19.

The incongruency between observational and self-reported parenting and that between performance-based and reported EF have raised questions about the generalization of findings in studies with only one measure for parenting or EF. Unfortunately, among the large number of studies on parenting and EF, very few studies used a multi-method approach to measure parenting or EF. In Valcan et al. (2018)’s meta-analysis study, they tried to conduct a moderation analysis of measurement methods on the relations between parenting and EF but failed due to the very limited number of studies. Results of some studies (Karreman et al., 2008; Zvara et al., 2019) indicated measurement methods might influence the relations between some aspects of parenting and some EF subcomponents. For example, Zvara et al. (2019) found that parents’ sensitivity and dyadic mutuality in the parent–child interactions were related to children’s performance in a hot EF task (delayed gratification) instead of parent-reported children’s behavioral problems with EF. Another study by Karreman et al. (2008) examined how measurement methods influenced the relationship between parenting and effort control (a self-regulatory aspect of temperament), and the results showed that the relationship was more robust when the same method (observational measures, reported measures) was used.

Therefore, the goal of this study was to examine the effect of the measurement methods on the relationship between maternal parenting behaviors and children’s EF among a group of Chinese preschoolers. Using a multi-method approach to measure maternal parenting (observational vs. self-reported) and children’s EF (performance-based vs. parent-reported), this study firstly investigated the agreement between different maternal parenting measures and that between children’s EF measures, and then examined how different measures of parenting were related to performance-based and parent-reported EF.

## 2. Method

### 2.1. Participants

One hundred and twenty-six Chinese preschoolers (64 girls; age range = 42–54 months, mean age = 48.65 months, SD = 3.58) and their mothers (age range = 25–44 years, mean age = 35.06 years, SD = 3.43; fathers’ age range = 25–48 years, mean age = 36.68 years, SD = 4.18) were recruited from three kindergartens in Shanghai, China. Letters of information were initially sent to the parents of about 150 children, which was calculated by the number of variables used in the hypothesized model using the 10-times rule (Hair et al., 2011). The children attended kindergarten in their first year and none of them were diagnosed with any cognitive, emotional, or behavioral disorders (based on the report of their teachers and mothers). Most parents had four-year university studies (36% fathers, 51% mothers) or graduate studies (33% fathers, 16% mothers), some of them had three-year college studies (19% fathers, 21% mothers), and the remaining had high school studies (13% fathers, 12% mothers). Parental consent and ethical approval from the authors’ affiliation were obtained before testing.

### 2.2. Materials

#### 2.2.1. Performance-based EF

Previous studies showed that as young as 3-year-old children may not be capable of switching from one task set to another (e.g., Chevalier and Blaye, 2008; Hanania, 2010), and some review studies (e.g., Garon et al., 2008) claimed that cognitive flexibility emerges after 3-year-old. Thus, we assessed only working memory and inhibition in our study.

*Working Memory*. Backward Digit Span (Wechsler, 1974) and Corsi Block-Tapping (adapted from Corsi, 1972) were used to assess children’s verbal and visual working memory, respectively. In Backward Digit Span, children were asked to reversely recall a sequence of digits that increased in number. In Corsi Block-Tapping, children were required to repeat the sequence of blue squares they have seen in reverse order. Both tasks consisted of 2–6 span with two trials in each span and were discontinued when children’s responses in both trials in each span were wrong. Both tasks have been used in previous studies to assess children as young as 3- to 4-year-old and showed good or acceptable reliability (e.g., Chen and Stevenson, 1988; Lehmann et al., 2014). Children’s score in each task was the total number of correct trails. Cronbach’s α reliability coefficient for two tasks was 0.80 and 0.78 in the present sample, respectively.

*Inhibition*. Day-Night Stroop (adapted from Gerstadt et al., 1994) and Head-Toes-Knees-Shoulders (HTKS; McClelland et al., 2014) were used to assess children’s inhibition. The Day-Night Stroop task required children to say “day” as soon as possible when seeing black cards displaying stars and the moon and to say “night” immediately when seeing white cards displaying the sun. The score was calculated by dividing the number of the total corrects out of 16 trials by the total time used to name the cards. HTKS required children to do the opposite of what they were told (e.g., to touch their toes when told to touch their head). Children were given 30 trials and were awarded 2 points for a correct response, 1 point for a self-corrected response, and 0 points for an incorrect response. The Cronbach’s α reliability coefficients for two tasks reported in McClelland et al. (2014) ranged from 0.92 to 0.99 for 3- to 6-year-old young children.

#### 2.2.2. Mother-rated EF

The Childhood Executive Functioning Inventory (Thorell and Nyberg, 2008) was used to measure four EF subcomponents: working memory (11 items; e.g., has difficulty remembering lengthy instructions), planning (4 items; e.g., has difficulty with activities that involve several steps), inhibition (6 items; has a tendency to do things without first thinking about what could happen), and regulation (5 items, has clear difficulties doing things he/she finds boring). Mothers rated their child on each item using a 5-point Likert scale ranging from 1 (definitely not true) to 5 (definitely true), with higher scores representing more behavioral difficulties in EF. The Cronbach’s α reliability coefficients for four scales in this study ranged from 0.74 to 0.92.

#### 2.2.3. Observational maternal parenting

The interactions between mothers and children were observed in two activities (free play and instructional activity; approximately 15 min for each activity). During free play, mothers and children were informed that they could play with provided toys; in the instructional activity, mothers were instructed to help children build a figure with Lego blocks. Maternal behaviors were coded from video recordings by four research assistants using the Parental Warmth and Control Scale (Rubin and Cheah, 2000). Two warmth aspects (positive emotion and positive sensitivity) and two control aspects (positive control and negative control) in the coding system were used. Positive emotion refers to behavioral and verbal expressions of happiness, comfort, connection, and warmth toward their children. Positive sensitivity indicates mothers’ ability to respond timely and appropriately to children’s verbal and non-verbal requests. Positive control represents the mothers’ promotion and scaffoldings of children’s behavior (e.g., the child had nothing to do, the mother verbally helped the child and explained the activity). Negative control refers to mothers’ behavior that was inappropriate and excessive controlling children’s behaviors (e.g., mothers grabbed toys from children to demonstrate the use of toys). The quality of interactions for each aspect of parenting was coded in 10 s epochs for all segments and rated on a 3-point Likert scale (0 = none, 1 = moderate, and 2 = outright/extensive), and the average scores were used in the analysis. Inter-rater consistency was assessed by independent recoding of about 20% of the interactions, and Cohen’s weighted kappa for four parenting aspects ranged from 0.81–0.93.

#### 2.2.4. Self-reported maternal parenting

The Parenting Styles Dimensions Questionnaire (Robinson et al., 2001) was used to measure maternal parenting. To conform with the aspects of observational parenting, this study used items of warmth and support (7 items; e.g., encourages child to talk about child’s problems), autonomy granting (4 items; e.g., allows child to give input into family rules), physical coercion (5 items; e.g., slaps child when the child misbehave), and verbal hostility (3 items; e.g., explodes in anger toward child). Mothers rated their behaviors in each item on a 5-point scale ranging from 1 (never) to 5 (always). The Cronbach’s α reliability coefficient of four parenting aspects in this study ranged from 0.62 to 0.73.

### 2.3. Procedure

Mothers and children were first asked to play freely with provided toys in a quiet room in children’s kindergartens, and then mothers were instructed to help their children in the instructive activity. The interactions were video-recorded by research assistants, who stayed beside the camera to adjust the angle and were required not to interfere with children or mothers. After the interaction, the children were individually tested by the research assistants in the same room, and the mothers completed questionnaires in another room. The recorded videos were coded following the coding scheme.

### 2.4. Statistical analyses

All the statistics were implemented with R software (R. Core Team, 2021). The preliminary data analysis was performed using “*psych”* package for R software (Revelle, 2022), and confirmatory factor analysis (CFA) and structural equation modeling (SEM) analysis were conducted using “*lavaan”* package (Rosseel, 2012). The normed Chi-square (*χ*^2^/df), the Comparative Fit Index (CFI), the Tucker–Lewis index (TLI), and the Root Mean Square Error of Approximation (RMSEA) were used to evaluate the model fit. The criteria of good fit used in this study are *χ*^2^/df < 2, CFI > 0.90, TLI > 0.90, and RMSEA < 0.08 (see Gana and Broc, 2019 for a summary).

## 3. Results

### 3.1. Preliminary data analyses

Table 1 presents descriptive statistics (mean and SD) for the measures in our study and the results of the Pearson correlation analysis (Bonferroni’s correction was applied). Firstly, the correlation for the parenting aspects assessed with two methods (observational vs. self-reported) was not significant. Secondly, except for the correlations of HTKS with observational positive control (*r* = 0.31, adjusted *p* < 0.05), none of the parenting aspects assessed by either method significantly correlated with children’s performance-based EF. Thirdly, none of the observational parenting aspects correlated significantly with mother-reported difficulties in any EF subcomponent. Regarding the relationship between parenting and children’s EF both assessed by mothers’ reports, *warmth and support* along with autonomy granting significantly correlated with planning and working memory.

**Table 1:** Means and standard deviations of the variables used in this study and their correlations.

Variable	*M*	SD	1	2	3	4	5	6	7	8	9	10	11	12	13	14	15	16
1. parent’s education	2.80	0.86																
2. positive emotion (O)	1.12	0.09	0.08															
3. positive control (O)	1.37	0.08	0.20	0.02														
4. negative control (O)	1.05	0.06	0.13	0.09	0.14													
5. positive sensitivity (O)	0.72	0.37	−0.01	0.28	0.02	0.15												
6. warmth and support (R)	4.25	0.44	0.21	0.01	0.11	0.00	0.02											
7. autonomy granting (R)	3.90	0.60	0.30	0.07	0.16	0.05	0.04	0.63*										
8. physical coercion (R)	1.89	0.49	−0.11	−0.11	−0.01	0.06	−0.09	−0.34*	−0.30									
9. verbal hostility (R)	1.93	0.59	−0.12	−0.17	0.07	−0.02	−0.15	−0.26	−0.25	0.65*								
10. HTKS (P)	36.76	15.17	0.32*	0.08	0.31*	0.30	−0.03	0.23	0.18	0.10	−0.10							
11. Digit WM (P)	1.78	1.35	0.23	0.07	0.18	0.17	−0.16	0.10	0.08	0.06	0.16	0.28						
12. Day-night Stroop (P)	0.43	0.22	0.08	−0.15	0.13	0.08	−0.06	0.07	0.03	−0.05	−0.07	0.24	0.36*					
13. Visual WM (P)	1.94	1.33	0.16	−0.06	0.18	0.17	−0.07	0.09	0.07	0.11	0.10	0.32*	0.41*	0.32*				
14. WM (R)	2.36	0.58	−0.16	−0.03	−0.04	−0.11	0.07	−0.40*	−0.39*	0.11	0.17	−0.21	−0.17	−0.10	−0.08			
15. planning (R)	2.41	0.61	−0.27	−0.09	0.00	−0.02	0.07	−0.32*	−0.35*	0.20	0.23	−0.10	−0.14	−0.05	0.01	0.84*		
16. regulation (R)	3.03	0.63	−0.05	0.07	0.00	0.07	0.02	−0.10	−0.14	0.09	0.15	−0.04	0.03	−0.06	0.10	0.63*	0.54*	
17. inhibition (R)	2.97	0.56	−0.19	0.00	−0.04	0.02	0.06	−0.16	−0.11	0.26	0.26	−0.03	0.05	−0.02	−0.01	0.54*	0.56*	0.59*

Parenting variables with (O) and (R) were observational and self-reported, respectively. EF variables with (P) and (R) were performance-based and mother-reported, respectively. WM = working memory. Bonferroni’s correction for multi tests was applied. *adjusted *p* < 0.05.

### 3.2. Results of SEM

Before running SEM, CFA was conducted for performance-based EF, since its four measures were adopted from different sources. Results showed that both one-factor model (EF) and two-factor model (working memory and inhibition) fit the data well (one-factor: *χ*^2^ = 0.14, df = 2, *p *= 0.93, *χ*^2^/df = 0.07, CFI = 1.00, TLI = 1.90, RMSEA = 0.00; two-factor: *χ*^2^ = 0.10, df = 1, *p* = 0.92, *χ*^2^/df = 0.10, CFI = 1.00, TLI = 1.10, RMSEA = 0.00), and insignificant difference was found between two models (Δ*χ*^2^ = 0.13, Δdf = 1, *p* = 0.72). Therefore, the more parsimonious model, i.e., one-factor performance-based EF, was used in the following predictive models.

SEM was then used to determine how maternal behaviors explained children’s EF. In the models, latent performance-based EF was loaded on Backward Digit Span, Corsi Block-Tapping, Day-Night Stroop, and HTKS, and latent reported EF (difficulties) was loaded on working memory, planning, regulation, and inhibition. Then different parenting aspects, along with parents’ education (averaged father’s and mother’s education level) as a covariate, were used to explain children’s EF. To simplify the model, two models were separately analyzed for observational and self-reported parenting, respectively.

**Figure 1:**
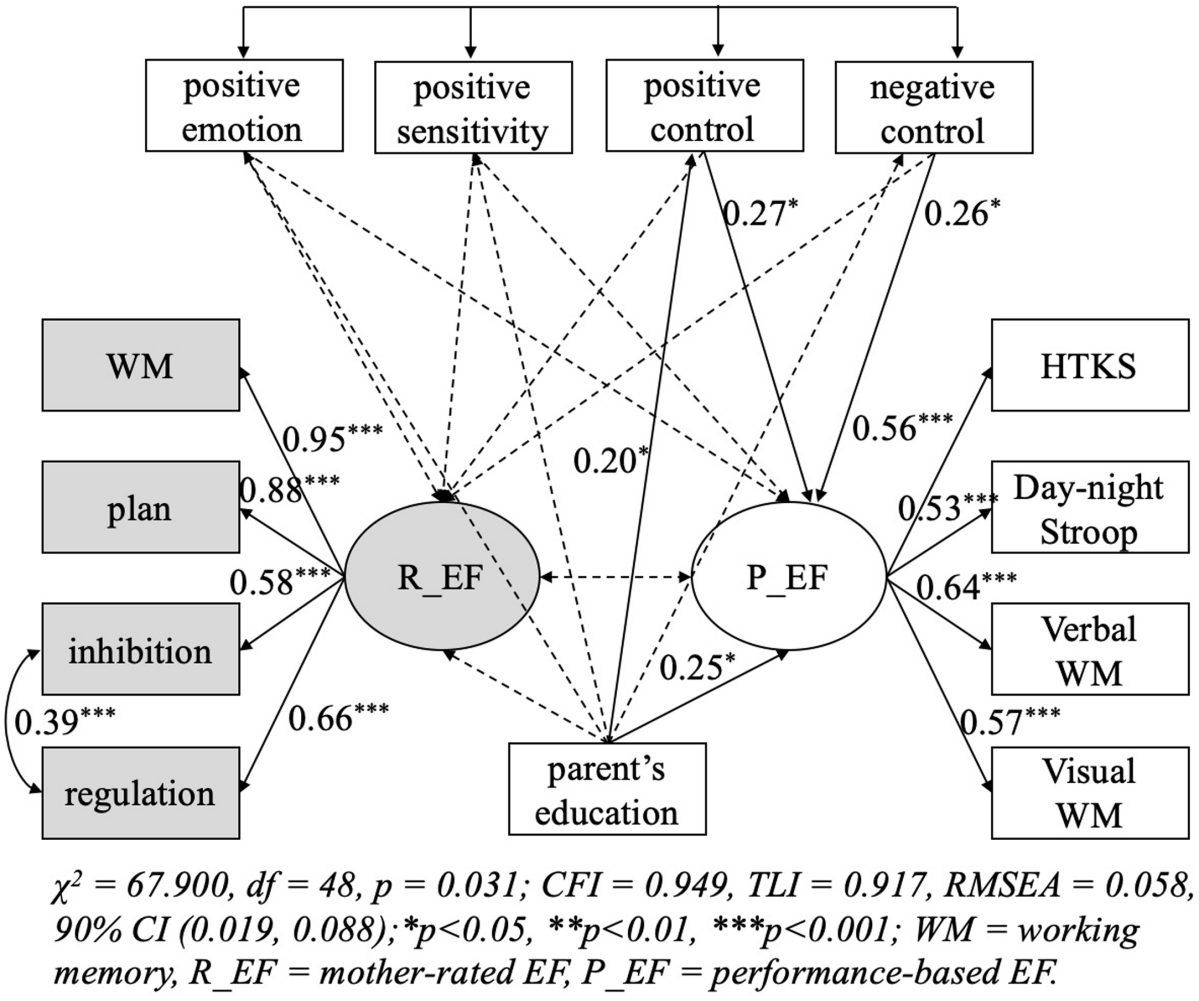
Predicting children’s executive functions from observational maternal parenting.

**Figure 2:**
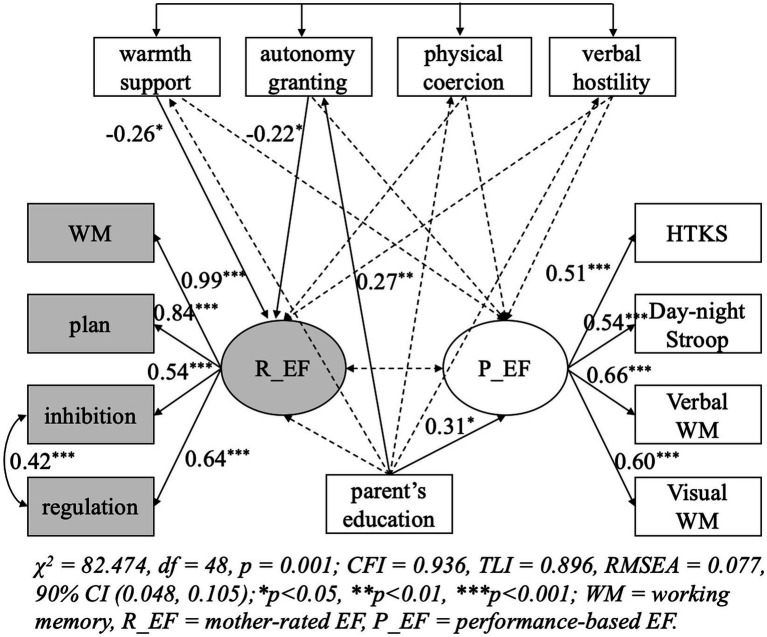
Predicting children’s executive functions from mother-reported parenting.

Neither the model for the observational nor self-reported parenting fitted the data well, and results of modification indices (MI) showed a correlation between the errors of regulation and inhibition (MI values = 17.45 and 18.05, respectively), which may indicate a specific relation between regulation and inhibition. After the modification, both models fitted the data well (the model for observational parenting, *χ*^2^ = 67.90, df = 48, *p* = 0.03, *χ*^2^/df = 1.41, CFI = 0.95, TLI = 0.92, RMSEA = 0.06; the model for self-reported parenting, *χ*^2^ = 82.47, df = 48, *p* = 0.001, *χ*^2^/df = 1.72, CFI = 0.94, TLI = 0.90, RMSEA = 0.08). Results of the model for observational and self-reported parenting are shown in Figures 1, 2, respectively. In the model for observational parenting, performance-based EF was positively predicted by positive control (*β* = 0.27, *p* < 0.05) and negative control (*β* = 0.26, *p* < 0.05). In the model for self-reported parenting, warmth and support (*β*= −0.26, *p*< 0.05) along with autonomy granting (*β*= −0.22, *p*< 0.05) negatively predicted mother-reported EF.

## 4. Discussion

Our results showed that no correlation was found between the observational and self-reported maternal parenting, nor between children’s performance-based and mother-reported EF. These findings were in line with previous studies using multiple measurement methods on parenting (e.g., Herbers et al., 2017; Zahidi et al., 2019) and EF (e.g., Buchanan, 2016; Saunders et al., 2018). The disagreement between self-reported and observational parenting may indicate they assess different parenting constructs. Compared with the measurement of actual behaviors in parent–child interactions, self-reported parenting may assess the perceptions, feelings, and attitudes of parenting behaviors (Gardner, 2000), and thus the parents’ subjective reports could be more influenced by social-desirability bias (Zahidi et al., 2019). Regarding the incongruence between performance-based and reported EF, two methods may also assess different aspects of EF. Performance-based EF may measure the cognitive aspect of EF and reflect the efficiency of information processing under the laboratory situations with minimal stimuli (Toplak et al., 2013). In contrast, reported EF may measure the behavioral and emotional aspects of EF and reflect the application of EF skills in daily situations surrounded by distracting stimuli (Anderson, 2002; Thorell and Nyberg, 2008).

The disagreement between different measures of parenting or EF did influence the relationship between maternal parenting and children’s EF in our results. Specifically, children’s performance-based EF was explained by only observational maternal parenting, while mother-rated EF was explained by only self-reported parenting. These results extended existing work (Karreman et al., 2008; Zvara et al., 2019) to general parenting and children’s general EF. The relationship patterns between maternal parenting and children’s EF with different measures would be discussed below.

### 4.1. Maternal parenting and performance-based EF

In the results, observational positive and negative control uniquely predicted performance-based EF. Previous studies showed that positive control such as scaffolding instead of warmth and support uniquely predicted children’s performance on EF tasks (Zaslow et al., 2006; Bernier et al., 2010), and one meta-analysis study by Karreman et al. (2006) also showed that children’s EF correlated higher with parental control than responsiveness. Mothers provided more scaffolding behaviors such as guiding and encouraging during everyday interaction with children, which would help children practice executive functions in goal-directed activities. However, the reported similar parenting construct, i.e., autonomy granting, did not predict the performance-based EF. One reason may be that positive control such as scaffolding was typically conceptualized as parenting skills and better assessed with observation method (Mermelshtine, 2017). Therefore, the observational maternal control may be more accurate than the self-reported maternal control, and only maternal positive and negative control in the interaction uniquely predicted performance-based EF.

Out of our expectation, negative control explained performance-based EF positively, which was at odds with previous studies (e.g., Halse et al., 2019; for a review, see Karreman et al., 2006). One reason is that the meaning of negative control in our study differed from these in others studies, and the degree may be more moderate. For example, negative control in other studies may include anger, harshness, punishment (Karreman et al., 2006), while negative control in our study refers to parental behaviors that are ill-timed or inappropriately controlling relative to what the child is doing (Rubin and Cheah, 2000). In addition, the negative control in our study is similar as *guan* (to train) parenting in Chinese culture, defined as being directiveness, excess controlling, and restricting children’s behavior (Chao, 1994; Stewart et al., 1998). Findings of previous studies showed that *guan* parenting positively influences children (e.g., Stewart et al., 1998, 2002). Similarly, negative control in this study positively influences children’s EF.

### 4.2. Maternal parenting and mother-rated children’s difficulties In EF

*Warmth and support* along with autonomy granting uniquely explained the parent-reported EF, in line with their closer relationship in previous studies (e.g., Speidel et al., 2020). Our results also showed that no observational parenting aspects uniquely explained children’s difficulties in EF. The reason may be that both reported parenting and children’s difficulties in EF assessed the global view of a variety of behaviors in everyday life over an extension periods of time (Thorell and Nyberg, 2008), while the observational parenting may reflect the mothers’ parenting behaviors only in specific contexts such as playing and teaching activities. Therefore, the daily family contexts of reported parenting behaviors may be shared with these of mother-rated children’s difficulties in EF.

Despite this, the closer relations between maternal parenting and children’s difficulties in EF may be inflated by the same reporter, i.e., the mother, who may tend to recall and report their parenting behavior management and children’s behaviors related to EF in the similar situations. Another reason may be the influence of the social-desirability bias (Zahidi et al., 2019). For example, parents may tend to report more frequent positive parenting behaviors and less negative parenting, and at the same time they may also report less children’s difficulties in EF.

### 4.3. Limitations and conclusion

The use of multiple measures of maternal parenting and children’s EF is a strength of this study, but some limitations should be mentioned. First, all the findings are concurrent and correlational and thus cannot imply the casual relationship between parenting and children’s EF. Second, although both observational and reported parenting include aspects related to responsiveness and control, it is hard to perfectly correspond the aspects of observational parenting with these of self-reported parenting due to the difference between their theoretical constructs. Future studies may consider unifying them to directly compare the agreement between two measures of the same parenting aspect. Third, paternal parenting was not included. Future studies can examine the role of paternal parenting since some studies showed that different aspects of maternal and paternal parenting explained children’s EF (e.g., Karreman et al., 2008). Fourth, we did not include measures on cognitive flexibility. Since EF has been typically conceptualized as a construct composed of working memory, inhibition, and cognitive flexibility (e.g., Miyake et al., 2000), future studies may include cognitive flexibility in their EF measures. Fifth, both maternal rated parenting and children’s rated EF were reported by mothers. Although some studies have shown that children’s EF reported by different observers (parents, teachers) were moderately correlated (e.g., Gutierrez et al., 2021), the performance-based EF correlated higher with children’s EF reported by their teachers than with that reported by their parents (e.g., Gutierrez et al., 2021). Future studies may use children’s information of multi-sources. Lastly, the mother–child interaction in our study was not recorded in natural settings (e.g., home), and mothers may behave differently when be observed (Gardner, 2000). Future studies may examine mothers’ parenting in home video recording.

Overall, the conclusion of this study is that the relationship between maternal parenting behaviors and children’s EF is influenced by measurement methods, and the relationship is more robust when the same/similar measurement is used. These findings have important implications for future studies on parenting and children’s EF. First, performance-based and parent-reported children’s EF may assess different aspects of EF, and observational and self-reported parenting may also measure different constructs of parenting behaviors. Therefore, future studies should consider both methods when examining the relations between EF/parenting and other predictors or outcomes. Second, since the relationship between parenting and children’s EF is influenced by the measurements of both parenting and EF, it should be cautious to directly compare their relationship across studies.

## Data availability statement

The datasets presented in this article are not readily available because some of the participants of this study did not agree for their data to be shared publicly. Requests to access the datasets should be directed to WW, vv_victorwei@126.com.

## Ethics statement

The studies involving human participants were reviewed and approved by Shanghai Normal University Ethics Committee. Written informed consent to participate in this study was provided by the participants’ legal guardian/next of kin.

## Author contributions

WW, M-MH, and YL: research design. WW and M-MH: data collection. WW, W-TL, and M-MH: data analysis. WW, W-TL, M-MH, and YL: manuscript writing. WW and YL: manuscript revising. All authors contributed to the article and approved the submitted version.

## Funding

This research was funded by the Ministry of Education in China Project of Humanities and Social Sciences (no. 18YJC880086).

## Conflict of interest

The authors declare that the research was conducted in the absence of any commercial or financial relationships that could be construed as a potential conflict of interest.

## Publisher’s note

All claims expressed in this article are solely those of the authors and do not necessarily represent those of their affiliated organizations, or those of the publisher, the editors and the reviewers. Any product that may be evaluated in this article, or claim that may be made by its manufacturer, is not guaranteed or endorsed by the publisher.
